# Comparative Evaluation of Serum Peroxiredoxin 2 (PRDX2), Serum Peroxiredoxin 4 (PRDX4), and Plasma Methylated Septin 9 (mSEPT9) Levels Against Conventional Biomarkers for Early Detection of Colorectal Cancer: A Study Protocol

**DOI:** 10.7759/cureus.111992

**Published:** 2026-07-03

**Authors:** Chetan K Kellellu, Subhashree Ray, Rajlaxmi Tiwari

**Affiliations:** 1 Department of Biochemistry, Institute of Medical Sciences and SUM Hospital, Siksha 'O' Anusandhan (Deemed to be University), Bhubaneswar, IND

**Keywords:** biomarkers, colorectal cancer, early detection, epigenetics, msept9, oxidative stress, prdx2, prdx4

## Abstract

Background: Colorectal cancer has been characterised as one of the major causes of morbidity and mortality due to cancer globally, and its load is increasing in India with a growing percentage of younger patients. Early intervention (by diagnosis) is associated with a substantial improvement in survival; however, current screening methods, such as colonoscopy, are expensive and invasive, and stool-based and conventional serum biomarkers demonstrate poor sensitivity to disease at an early stage. Oxidative stress and epigenetic modifications play a key role in colorectal carcinogenesis, and circulating indicators of these pathways can enhance non-invasive early diagnosis. The objective of this study is to compare the diagnostic accuracy of serum peroxiredoxin 2 (PRDX2), serum peroxiredoxin 4 (PRDX4), and plasma methylated septin 9 (mSEPT9) with conventional biomarkers for the early detection of colorectal cancer in an Indian population.

Methods: This hospital-based case-control study will include 100 participants, including 50 histopathologically confirmed untreated early-stage colorectal cancer patients (stage I-II) and 50 age- and sex-matched healthy controls without malignancy or chronic inflammatory disease, based in a hospital. The sample size was determined based on the expected difference in diagnostic performance between novel and conventional biomarkers, as described in the Methods section. Venous blood will be gathered after overnight fasting and processed to get serum and plasma, which will be stored at −80°C until analysis. The study has received institutional ethics committee (IEC) approval. The levels of serum PRDX2, serum PRDX4, plasma mSEPT9, carcinoembryonic antigen 19-9, C-reactive protein, neutrophil gelatinase-associated lipocalin, interleukin-6, and estimated glomerular filtration rate will be determined either with standardised enzyme-linked immunosorbent assay procedures or with computation formulas. To evaluate individual and multi-markers, the receiver operating characteristic (ROC) curve will be used to evaluate the diagnostic performance, and multivariate logistic regression models will be used to evaluate the diagnostic performance of individual and multi-markers. The primary outcome will be the diagnostic accuracy of PRDX2, PRDX4, and mSEPT9 assessed using the area under the ROC curve, while secondary outcomes will include sensitivity, specificity, positive predictive value, negative predictive value, and the diagnostic performance of combined biomarker panels.

Discussion: This protocol describes a systematic assessment of both oxidative stress and epigenetic biomarkers in addition to conventional biomarkers for the early detection of colorectal cancer in an Indian population. The study aims to improve the diagnostic sensitivity for early-stage colorectal cancer while maintaining feasibility for routine clinical laboratory practice by evaluating a minimally invasive blood-based panel comprising PRDX2, PRDX4, and mSEPT9. The findings of this study may provide preliminary evidence for the development of cost-effective, non-invasive screening strategies suitable for resource-limited settings and may support future validation studies and implementation research.

## Introduction

Colorectal cancer is a significant health issue and one of the most commonly diagnosed malignancies across the globe, and a primary cause of cancer-induced deaths. The worldwide cancer statistics represent that colorectal cancer is the third most diagnosed cancer and the second cause of cancer mortality, whereby the occurrence is expected to escalate significantly in the next decades [1-3]. The stage of diagnosis is a major determinant of prognosis, with five-year survival being over 90% when the disease is localised but less than 15% when it is at distant metastases, which has a crucial role in the importance of early diagnosis measures [3].

In India, a steady increase in cases of colorectal cancer has been noted, especially in the urban and fast-developing states, even though the overall incidence has been relatively low compared to most parts of the West. Lifestyle changes, which involve decreasing intake of dietary fibre, intake of processed foods, and lack of physical exercise, are identified as the main factors [4-6]. National cancer registries and regional hospital-based studies, such as those in Odisha, indicate an increasing disease burden of gastrointestinal malignancies with an increasingly higher ratio of colorectal cancer cases being reported at relatively younger ages [7-9].

Existing screening and diagnostic methods used to detect colorectal cancer are limited, especially in the low- and middle-income context. Colonoscopy is still the diagnostic gold standard, as it can be used to diagnose as well as treat; however, it is an invasive procedure, expensive, requires special infrastructure, and is not widely available to allow its usage to cover the entire population. Stool-based screening methods, such as faecal occult blood test (FOBT) and faecal immunochemical test (FIT), are non-invasive but have poor sensitivity to disease at an early stage and are subject to pre-analytical and compliance-related variables [10-12]. Traditional serum biomarkers (carcinoembryonic antigen and carbohydrate antigen 19-9) are not sensitive and specific enough to be applied in early screening and are mainly employed in prognostic testing and surveillance of treatment after its administration [13].

Epigenetic regulation and oxidative stress have significant roles in colorectal carcinogenesis. The peroxiredoxins are a group of antioxidant enzymes that are used in the management of reactive oxygen species and cellular redox homeostasis. Experimental and clinical studies have demonstrated that serum peroxiredoxin 2 (PRDX2) and serum peroxiredoxin 4 (PRDX4) are significantly upregulated in colorectal cancer and are associated with tumour progression and poor clinical outcomes, supporting their evaluation as circulating biomarkers [14-18]. These findings support their potential utility as circulating biomarkers reflective of tumour-associated oxidative stress. PRDX2 and PRDX4 were selected because they represent complementary components of the antioxidant defence system and have been independently associated with colorectal cancer progression, oxidative stress regulation, and poor clinical outcomes. Their combined evaluation may provide a more comprehensive assessment of tumour-associated oxidative stress than either biomarker alone. Recent experimental studies have further demonstrated that PRDX2 promotes colorectal cancer cell proliferation through modulation of the p53 pathway and is associated with poor prognosis, while comprehensive analyses have confirmed the prognostic significance of the PRDX family in colon adenocarcinoma. These findings further support the evaluation of PRDX2 and PRDX4 as potential circulating biomarkers for colorectal cancer [17,18].

In parallel, epigenetic biomarkers have emerged as promising tools for non-invasive colorectal cancer detection. Methylated septin 9 (mSEPT9) represents circulating tumour DNA arising from aberrant promoter hypermethylation of the SEPT9 gene and is detectable in plasma. Large prospective studies and meta-analyses have demonstrated the diagnostic utility of plasma mSEPT9, leading to its regulatory approval as a blood-based screening assay for colorectal cancer; however, its sensitivity for early-stage disease remains moderate [19,20]. Despite encouraging findings, previous studies have largely evaluated oxidative stress biomarkers and epigenetic biomarkers independently. Comparative studies assessing PRDX2, PRDX4, and mSEPT9 simultaneously alongside conventional biomarkers are limited, particularly in the Indian population. This represents an important knowledge gap that the present study aims to address.

Based on these observations, we hypothesise that the combined assessment of oxidative stress biomarkers (PRDX2 and PRDX4) and the epigenetic biomarker mSEPT9 will demonstrate superior diagnostic performance compared with conventional biomarkers for the early detection of colorectal cancer. Integrating oxidative stress-associated proteins and epigenetic biomarkers into a multi-marker blood-based panel may improve early detection performance while maintaining feasibility for routine clinical laboratories, particularly in resource-limited settings where minimally invasive and affordable diagnostic approaches are needed. The present hospital-based observational case-control study is therefore designed to systematically assess the diagnostic performance of these emerging biomarkers for early detection of colorectal cancer in an Indian setting.

## Materials and methods

Study aim, design, and setting

This study is designed to evaluate the diagnostic performance of serum PRDX2, PRDX4, and plasma mSEPT9 for the early detection of colorectal cancer. The primary outcome is the diagnostic accuracy of serum PRDX2, serum PRDX4, and plasma mSEPT9, assessed using the area under the receiver operating characteristic (ROC) curve (AUC). Secondary outcomes include sensitivity, specificity, positive predictive value (PPV), negative predictive value (NPV), and the diagnostic performance of combined biomarker panels. It is a hospital-based observational case-control study that will be carried out at an eastern Indian tertiary care teaching hospital. In order to evaluate the utility of biomarkers within the most essential diagnostic approach, the investigation's primary objective is to focus on the early stages of the disease.

Study population and eligibility criteria

Cases

The cases will consist of adult patients aged between 30 and 75 years of age with biopsy- or surgical specimen-proven cases of colorectal cancer. Tumours will be graded by the WHO Classification of Tumours of the Digestive System, and staging will be done using the American Joint Committee on Cancer (AJCC) tumour-node-metastasis staging system (8th edition) [21,22]. This will only include patients whose disease is in the early stage (stages I-II), so as to better concentrate on early detection and prevent confounding factors associated with advanced disease biology or treatment. Previous surgery, chemotherapy, radiotherapy, or targeted therapy will be excluded so as to avoid changes in circulating biomarker levels caused by treatment.

Controls

The controls will include age- and sex-matched healthy volunteers with no history of malignancy, inflammatory bowel disease, chronic inflammatory disease, or acute infection during the enrolment period. Individuals with diabetes mellitus, smoking history, alcohol consumption, obesity, metabolic syndrome, chronic liver disease, autoimmune disorders, hereditary colorectal cancer syndromes, previous malignancy, or any other condition known to influence oxidative stress biomarkers will be excluded. Information regarding the family history of colorectal cancer will also be collected and documented. Healthy controls will undergo appropriate clinical evaluation to exclude suspected colorectal malignancy before enrolment.

Sample collection and processing

Following an overnight fast, approximately 5 mL of venous blood will be collected from each participant into plain and EDTA (ethylenediaminetetraacetic acid) vacutainers. Samples will be centrifuged at 3,000 rpm for 10 minutes within one hour of collection. To minimise inter-assay variability, serum and plasma aliquots will be stored at -80°C until batch analysis.

Biomarker measurement

Serum PRDX2 and PRDX4 levels will be measured using commercially available sandwich enzyme-linked immunosorbent assay (ELISA) kits (PRDX2: Cloud-Clone Corp., Katy, TX; PRDX4: Elabscience, Wuhan, China), supplied by KRISHGEN Biosystems, Mumbai, India, and will be performed according to the manufacturers' instructions. Plasma mSEPT9 levels will be determined using a quantitative methylation-specific ELISA kit. The selected ELISA-based assay for mSEPT9 has been validated by the manufacturer for the quantitative measurement of mSEPT9 and will be performed according to the manufacturer's instructions. Conventional biomarkers, including carcinoembryonic antigen (CEA), carbohydrate antigen 19-9 (CA19-9), C-reactive protein (CRP), interleukin-6 (IL-6), and neutrophil gelatinase-associated lipocalin (NGAL), will also be measured using validated ELISA-based assays, where applicable. All biomarker assays will be performed according to the manufacturers' instructions. Quality control procedures, including calibration and internal quality control samples, will be followed throughout the study. Biomarker measurements will be performed in duplicate, wherever applicable, to minimise analytical variability.

Justification of biomarker selection

Peroxiredoxins play a major role in oxidative stress and redox signal transduction and have been involved in the pathogenesis of colorectal cancer in terms of tumour cell survival, proliferation, and resistance to oxidative stress. Experimental and clinical work conducted ahead of time has shown that PRDX2 and PRDX4 are significantly upregulated in colorectal cancer, which justifies their analysis as blood biomarkers [14-18]. The prospective validation as a blood-based screening biomarker of colorectal cancer is of mSEPT9, which is a circulating epigenetic modification product of tumour-specific DNA methylation alterations; the sensitivity of mSEPT9 in detecting early-stage disease is still suboptimal [19,20].

Sample size estimation

The sample size was calculated based on the expected diagnostic accuracy of the proposed biomarkers for ROC curve analysis, assuming a two-sided α = 0.05 and 80% statistical power. A total sample of 50 cases and 50 controls was considered adequate for this exploratory diagnostic accuracy study and is consistent with established methodological recommendations for biomarker evaluation [22-24].

Statistical analysis

Based on the distribution, summarisation of continuous variables will be done with the mean and standard deviation or the median with interquartile range. Student's t-test or Mann-Whitney U test will be used to make comparisons between cases and controls accordingly. The diagnostic performance will be determined through the analysis of the ROC curve, and the sensitivity, specificity, PPV, and NPV will be calculated. A combination of biomarkers will be evaluated using multivariable logistic regression models. ROC curves will be compared using appropriate statistical methods. Missing data, if encountered, will be assessed before analysis and handled using appropriate statistical techniques. The statistical calculations will be conducted by using the standard statistical software, and the statistical significance of the results will be taken as a p-value below 0.05. All statistical analyses will be performed using SPSS version 26 (IBM Corp., Armonk, NY).

Ethical considerations

The Institutional Ethics Committee of the Institute of Medical Sciences (IMS) and SUM Hospital, Siksha ‘O’ Anusandhan (Deemed to be University), Bhubaneswar, has approved the study (IEC/IMS.SH/SOA/2026/1212). All participants will be enrolled after obtaining written informed consent. The research will be performed in light of the principles of the Declaration of Helsinki. As this is an observational case-control study without therapeutic intervention, Clinical Trials Registry-India (CTRI) registration is not mandatory.

Strengths and limitations

The strengths of this study include the simultaneous evaluation of oxidative stress and epigenetic biomarkers alongside conventional biomarkers in early-stage colorectal cancer. Potential limitations include the single-centre design, moderate sample size, and possible selection bias inherent to hospital-based case-control studies.

## Results

Current study status

As this manuscript presents a study protocol, no study results are available at the time of submission. Ethical approval has been obtained, and the study procedures for participant recruitment, sample collection, laboratory analysis, and data management have been established. Participant enrolment and data collection will be conducted according to the approved study protocol. The findings of this study will be reported in a subsequent publication upon completion of data collection and analysis.

## Discussion

This protocol highlights several practical and operational issues associated with implementation in a resource-constrained, hospital-based environment in India. Recruitment of histopathologically confirmed untreated stage I-II colorectal cancer patients may be challenging because many patients present with more advanced disease. Careful participant selection and documentation of potential confounding factors will therefore be important to ensure the validity of the study findings [3,4]. Previous studies have evaluated oxidative stress biomarkers and epigenetic biomarkers independently for colorectal cancer detection. However, limited evidence is available regarding the combined diagnostic performance of PRDX2, PRDX4, and plasma mSEPT9 alongside conventional biomarkers, particularly in the Indian population. The present protocol addresses this knowledge gap by proposing a standardised multimarker approach for the early detection of colorectal cancer. Future multicentre prospective studies will be required to externally validate the diagnostic performance and generalisability of the proposed biomarker panel. The proposed multimarker strategy integrates complementary biological pathways by simultaneously evaluating oxidative stress-associated proteins (PRDX2 and PRDX4) together with tumour-specific epigenetic alterations (mSEPT9). Such an approach may improve diagnostic discrimination compared with single-biomarker strategies and provide a stronger biological rationale for multi-biomarker-based colorectal cancer screening.

Standardised laboratory procedures, including appropriate sample handling, storage, and quality control measures, are essential to minimise pre-analytical and analytical variability during biomarker estimation. Careful laboratory workflow planning will also be necessary to ensure reliable and reproducible biomarker measurements [15-18].

If validated, the proposed blood-based biomarker panel may complement existing colorectal cancer screening strategies by providing a minimally invasive approach suitable for routine clinical laboratories. Such an approach may improve access to early detection, particularly in resource-limited settings where colonoscopy-based screening is not readily available. If successfully validated, the proposed biomarker panel may offer a clinically feasible turnaround time compatible with routine diagnostic laboratory workflows. Furthermore, the use of blood-based biomarkers has the potential to reduce healthcare costs associated with invasive diagnostic procedures, although formal cost-effectiveness analyses will be required in future studies. From a methodological perspective, the case-control design is highly effective when used to conduct an exploratory diagnostic assessment, but it can be overly optimistic about diagnostic performance when compared with actual screening environments where disease prevalence is lower and spectrum bias is greater. Histopathological examination will serve as the reference standard for confirming colorectal cancer in cases. Despite careful participant selection and predefined eligibility criteria, the possibility of verification bias, an inherent limitation of hospital-based case-control studies, cannot be completely excluded. Although the sample size provides adequate statistical power to detect the anticipated difference in diagnostic performance, it may not be sufficient for extensive subgroup analyses; therefore, such analyses should be considered exploratory and interpreted with caution [24,25].

Publication of this protocol promotes methodological transparency, minimises selective reporting bias, and provides a standardised framework for future multicentre validation studies evaluating multimarker panels for colorectal cancer screening in India. The findings from future validation studies may support the integration of promising biomarker panels into colorectal cancer screening strategies, particularly in resource-limited settings [24,25].

## Conclusions

This protocol outlines a systematic approach to evaluating novel oxidative stress and epigenetic biomarkers for the early detection of colorectal cancer. The study is designed to evaluate the diagnostic performance of a minimally invasive biomarker panel comprising PRDX2, PRDX4, and plasma mSEPT9 in comparison with conventional biomarkers in an Indian population. The findings of this study may provide preliminary evidence to support future validation studies and the development of improved colorectal cancer screening and early diagnostic strategies.

## Figures and Tables

**Figure 1:**
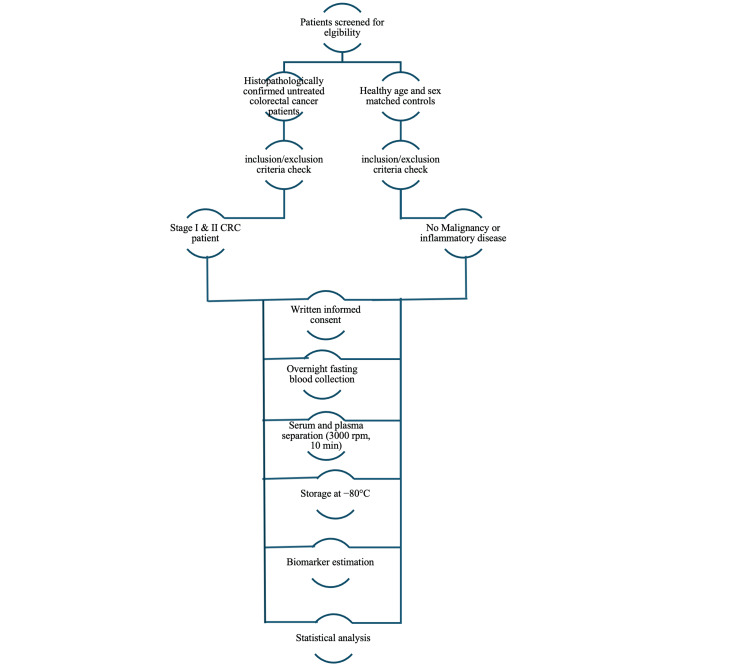
Study flowchart. CRC: colorectal cancer.

## Appendices

**Table 1:** Biomarker panel. PRDX2: peroxiredoxin 2; PRDX4: peroxiredoxin 4; mSEPT9: methylated septin 9; CEA: carcinoembryonic antigen; CA19-9: carbohydrate antigen 19-9; CRP: C-reactive protein; IL-6: interleukin 6; NGAL: neutrophil gelatinase-associated lipocalin; eGFR: estimated glomerular filtration rate; ELISA: enzyme-linked immunosorbent assay; CKD-EPI: Chronic Kidney Disease Epidemiology Collaboration.

Biomarker	Sample	Method	Purpose
PRDX2	Serum	ELISA	Primary biomarker
PRDX4	Serum	ELISA	Primary biomarker
mSEPT9	Plasma	Quantitative methylation-specific ELISA	Primary biomarker
CEA	Serum	ELISA	Comparator
CA19-9	Serum	ELISA	Comparator
CRP	Serum	ELISA	Secondary biomarker
IL-6	Serum	ELISA	Secondary biomarker
NGAL	Serum	ELISA	Secondary biomarker
eGFR	Serum	CKD-EPI equation	Secondary parameter

**Table 2:** Study timeline and phases of the research protocol.

Phase	Duration	Activity
I	Jan–Mar 2026	Ethical clearance, kit procurement, participant recruitment
II	Apr–Dec 2026	Sample collection and biomarker analysis
III	Jan–Feb 2027	Data compilation and statistical analysis
IV	Feb–Apr 2027	Manuscript preparation and submission
